# Osteocalcin levels decrease during the treatment of an acute depressive episode

**DOI:** 10.3389/fpsyt.2022.893012

**Published:** 2022-08-02

**Authors:** Elis Bartečků, Jana Hořínková, Pavel Křenek, Alena Damborská, Josef Tomandl, Marie Tomandlová, Jan Kučera, Jana Fialová Kučerová, Julie Bienertová-Vašků

**Affiliations:** ^1^Department of Psychiatry, Faculty of Medicine, University Hospital Brno, Masaryk University, Brno, Czechia; ^2^Department of Biochemistry, Faculty of Medicine, Masaryk University, Brno, Czechia; ^3^RECETOX, Faculty of Science, Masaryk University, Brno, Czechia; ^4^Department of Pathological Physiology, Faculty of Medicine, Masaryk University, Brno, Czechia

**Keywords:** osteocalcin, major depressive disorder, biomarker, depression, antidepressant treatment

## Abstract

**Objectives:**

Osteocalcin is a protein secreted by osteoblasts with a versatile endocrine role. Several domains in which it plays a role—stress response, monoamine synthesis, and cognitive functioning—are implicated also in the pathophysiology of major depressive disorder. In search of possible objective biomarkers of depression, the aim of the study was to assess the relationship between osteocalcin and depressive symptoms during the treatment of depressive episode.

**Methods:**

The study included female inpatients with at least moderate depressive episode. In these patients, depression severity was measured using the Montgomery-Åsberg Depression Rating Scale (MADRS), and osteocalcin levels were assessed before the stabilization of antidepressive treatment and after 6 weeks. Relationships between osteocalcin levels and symptoms were analyzed with mixed-effect and linear models, taking into account age, menopausal status, and body mass index.

**Results:**

In 11 out of 13 enrolled inpatients, osteocalcin levels decreased during the first 6 weeks of treatment; this decrease was significant according to the mixed-effects model (*t* = −2.345, *p* = 0.019). According to the linear model, this decrease was significantly associated with reduction in depressive symptom severity (*t* = 2.673, *p* = 0.028). Osteocalcin was not associated with initial depressive symptom severity, and initial osteocalcin levels did not predict response to treatment. Limitations of the study include low sample size and inclusion of both pre- and postmenopausal women of various ages.

**Conclusions:**

This preliminary study suggests that osteocalcin may be a candidate biomarker of antidepressive treatment response and that this topic warrants further investigation.

## 1. Introduction

Osteocalcin is a protein secreted by osteoblasts with a suggested versatile endocrine role in the regulation of metabolism, adaptation to exercise, male fertility, and neuronal development (1).

The variety of its possible roles is reflected by numerous factors affecting its blood levels. Osteocalcin synthesis is K-vitamin dependent (2). It is influenced by ethnicity (3), age, sex and menopausal status in women. For example, young adult men have higher levels than young women of the same age, then levels decrease and remain stable in middle-aged of both sexes; in females, it increases when transitioning to menopause (4). Its connection to sex hormones is further signified by the observation that oral contraceptives and hormone replacement therapy after menopause are associated with lower osteocalcin levels (4).

Exercise leads to an increase in osteocalcin levels (5, 6), and, in contrast, osteopenia and osteoporosis are often mentioned to be associated with its decrease (7). On the other hand, a recent meta-analysis found no difference in serum osteocalcin levels between postmenopausal women with and without osteoporosis (8).

Osteocalcin secretion is regulated by insulin, and there is a feed-forward loop leading to increased proliferation of pancreatic β-cells (9). Subsequently, body mass index (BMI) and percentage body fat show a negative correlation with osteocalcin levels (10). Metabolic syndrome and related variables (waist circumference, blood pressure, fasting plasma glucose, HbA1c, uric acid, alkaline phosphatase, C-reactive protein levels) are associated with decreased levels of osteocalcin independently of postmenopausal status (11).

Apart from these connections, evidence points to the involvement of osteocalcin in stress response and functions of the central nervous system. It has been suggested that the acute stress reaction is associated with an increase in osteocalcin levels, which is probably mediated by increased glutamate uptake by osteoblasts (12). Moreover, osteocalcin acts as a neuroactive substance. Especially in its undercarboxylated form, it crosses the blood-brain barrier (13), binds to the neurons of the brain stem, midbrain, and hippocampus, and regulates the genes that are important for monoamine (increase) and GABA (decrease) syntheses (1). Interestingly, in animal models of anxiety and depression, osteocalcin deficient mice show more behaviors associated with anxiety and depression, deficits in spatial learning, and decreased adult neurogenesis in the hippocampus (1).

The role of osteocalcin in stress response, monoamine synthesis and neurogenesis—domains implicated in the pathophysiology of major depressive disorder (14)—suggests that this protein may be directly or indirectly associated with the pathogenetic pathways of depression and may have value as a biomarker.

This putative connection was studied mostly indirectly in the context of bone mineralization deficits in women with major depressive disorder. Several studies found decreased osteocalcin in patients with past or current depression (15) and in patients treated for depression (16, 17). Other studies found increased osteocalcin levels in patients with major depressive disorder (18, 19) or no difference between patients and controls (20). Interestingly, in one study, osteocalcin plasmatic level was positively associated with subjectively perceived stress in patients with depression (21).

The literature search yielded only a single prospective study exploring osteocalcin changes during the treatment of depression by Aydin et al., in which osteocalcin increased during the first 3 months of treatment with escitalopram in 47 women with the first episode of major depressive disorder; interestingly, before treatment, osteocalcin was significantly positively correlated with depression severity (22).

Presented evidence suggests that osteocalcin could be a biomarker of depression severity and treatment response. However, the evidence is limited, and it is unclear if existing findings warrant this interpretation. The aim of this study was to assess the utility of osteocalcin as a biomarker of depressive episode severity and its changes during treatment in patients with an acute depressive episode of at least medium severity.

## 2. Materials and methods

### 2.1. Subjects

The study included hospitalized women aged 25–70 years, with major depressive disorder, with a current episode of at least moderate severity, who signed informed consent. Episode severity was limited to moderate and severe in order to increase the homogeneity of the sample and reduce the risk of diagnostic error.

Exclusion criteria were: known organic disorder influencing brain function, other concurrent acute psychiatric disorder with the exception of personality disorders and substance abuse (past psychiatric diagnosis was not an exclusion criterion), intellectual disability, current use of immunosuppressive or immunomodulatory medication, pregnancy, uncooperativeness, and deprivation of legal capacity. Furthermore, no patients with diseases or treatment known to significantly influence bone metabolism were enrolled.

The study has been carried out in accordance with The Code of Ethics of the World Medical Association (Declaration of Helsinki) for experiments involving humans. Informed consent was obtained for all subjects before enrollment into the study. The study was approved by the institutional ethics committee.

### 2.2. Clinical variables

The diagnosis was established using the criteria of the Diagnostic and Statistical Manual of Mental Disorders (DSM-5) (23). Information about past comorbidities was gathered from patient documentation which used diagnoses according to the International Classification of Diseases, 10th edition.

The clinical state was assessed twice: the week after admission, i.e., before the stabilization of therapy (Week 0, W0), and 6 weeks later (Week 6, W6). The severity of depressive symptoms was assessed using the Montgomery-Åsberg Depression Rating Scale (MADRS) (24).

This was strictly naturalistic observational study. Patient treatment was based on clinical judgment and was not affected by study inclusion. Because patients used different classes of medication (see Table 1), treatment was described with a custom ordinal scale, further referred to as “medication index”: 0 - no medication, 1 - one or more drugs in lower than the therapeutic dose, 2 - one drug in the therapeutic dose, 3 - more drugs with one in the therapeutic dose, 4 - more drugs in the therapeutic dose. The highest achieved medication index during the study was considered. Therapeutic doses of antidepressants were established according to the Antidepressant Treatment History Questionnaire (ATRQ) (25) and antipsychotics according to Wang et al. (26). Medications not listed in the ATRQ, in the review by Wang et al. or not indicated for the treatment of depression according to the Product Characteristics Summary, were not used to calculate the medication index.

**Table 1:** Number of patients treated by each medication.

**Compound**	**Type**	**W0**	**W6**	**Compound**	**Type**	**W0**	**W6**
Citalopram	AD	2	2	Escitalopram	AD	0	1
Sertraline	AD	1	2	Venlafaxine	AD	3	2
Mirtazapine	AD	0	1	Trazodone	AD	1	1
Vortioxetine	AD	2	3	Agomelatine	AD	2	3
Clomipramine	AD	0	1	Clozapine	AP	0	1
Olanzapine	AP	3	4	Quetiapine	AP	3	3
Amisulpride	AP	3	3	Tiapride	AP	2	0
Aripiprazole	AP	2	1	Lithium	MS	0	1
Carbamazepine	MS	1	0	Pregabalin	Other	1	2
Clonazepam	Adjuvant	5	1	Oxazepam	Adjuvant	4	4
Zolpidem	Adjuvant	1	0	Promethazine	Adjuvant	1	0

*AD, antidepressants; AP, antipsychotics; MS, mood stabilizers*.

### 2.3. Osteocalcin levels

The quantitative measurement of undercarboxylated osteocalcin in plasma samples was performed using a commercially available enzyme immunoassay kit (#MBS700581, MyBioSource, CA, USA) according to the manufacturer's instructions.

### 2.4. Statistical analysis

Normality of the distributions of quantitative variables was tested with the Shapiro-Wilk normality test (*p* > 0.05 for normal distribution). Because of the low sample size, only three tests were conducted: The relationships between absolute MADRS and osteocalcin levels during the treatment were analyzed using a mixed-effect model (model 1); the relationships between MADRS and osteocalcin changes (model 2) and the relationships between MADRS change and osteocalcin level in W0 (model 3) were analyzed with linear models. In the first (mixed-effects) model, absolute values of MADRS and osteocalcin were analyzed. In the second and third models, changes in MADRS and osteocalcin changes were expressed as percent changes of the values in W0. This approach ensured that both absolute and relative changes were assessed. In all models, three covariates were used: age, menopausal status and BMI. These variables were selected to cover the most important factors influencing osteocalcin levels. More covariates, for example, factors associated with metabolic syndrome, were not included considering the small sample size, probable multicollinearity and to reduce the risk of model overfitting. Medication was not used in models because of a relative homogeneity of treatment strategies observed in the study (see below) and because the somatic medication was stable during the 6 weeks of observation. A *p*-value lower than 0.05 was considered significant. All the models underwent basic diagnostics to ensure the normal distribution of residuals - visual inspection of distributions of residuals and the Shapiro-Wilk normality test. Furthermore, partial η^2^ was used as a measure of the effect size of each independent variable (27). The analysis was performed with the R Project for statistical computing, version 4.0.5 (28).

## 3. Results

### 3.1. Sample characteristics

In total, 13 subjects were enrolled, all females of the same Central European ethnic group. Five enrolled patients were in postmenopause during the study. There was no statistically significant difference in osteocalcin levels between patients with different menopausal status [osteocalcin levels in both time points, Welch Two Sample *t*-test: *t*_(17.669)_ = –1.1787, *p* = 0.254]. Although age and BMI varied between subjects, there was not a statistically significant correlation between osteocalcin levels and age [Pearson's correlation: *r*_(24)_ = 0.171, *p* = 0.403] or between osteocalcin levels and BMI [Pearson's correlation: *r*_(24)_ = 0.018, *p* = 0.932]. No patients with diagnosed osteoporosis, osteopenia, or using hormonal contraception or hormonal replacement therapy were enrolled in our study.

Eight patients were admitted after relapsing while using long-term maintenance antidepressant treatment; two patients were not administered any medication before admission. The remaining three patients were using medication for one, four and more than 6 weeks before the admission. Although, during the study, patients used various psychiatric drugs and doses, psychiatric treatment, as described by a medication index and medication groups (antidepressants, antipsychotics, mood stabilizers, other), was relatively homogenous: During the study, augmentation strategies were used in all but one patient, with the most common combination being antidepressants with antipsychotics in sufficient doses. Subsequently, in all but one patient, the maximal achieved medication index was equal to the maximum of the defined scale. Relevant patients characteristics are summarized in Table 2.

**Table 2:** Sample characteristics.

		**Range**	**Mean (SD)**	**Median (IQR)**
	Age	27–66	49.923 (12.325)	51 (17)
	BMI	17.374–30.078	24.547 (4.513)	25.352 (8.955)
Week 0	MADRS	22–52	32.231 (8.428)	32 (13)
	Osteocalcin [ng / ml]	3.03–8.01	4.872 (1.659)	5 (2.04)
Week 6	MADRS	2–36	12.923 (10.004)	13 (10)
	Osteocalcin [ng / ml]	2–6.79	4.257 (1.451)	4.25 (1.22)
Change	MADRS [%]	–91.892 - –20.588	–60.459 (26.144)	–65 (49.001)
	Osteocalcin [%]*	–33.993 - 40.789	–10.558 (24.08)	–18.772 (10.059)
**Number of experienced depressive episodes (including current episode)**				
Single episode	More episodes
4 (30.77%)	9 (69.233%)
**Number of previous hospitalizations**				
0	2	4–6	7–8
4 (30.77%)	4 (30.77%)	3 (23.08%)	2 (15.38%)
**Severity of current episode**				
Moderate	Severe	Psychotic
3 (23.08%)	10 (76.92%)	5 (38.46%)
**Comorbidities and diagnoses in the past**				
Single	Multiple	One of F131, F230, F432, F531 or F601	F412
3 (23.08%)	2 (15.38%)	1 (7.69%)	3 (23.08%)
**Treatment**				
AD	AP	MS	Other	ECT
13 (100%)	12 (92.31%)	2 (15.38%)	2 (15.38%)	1 (7.69%)
**Drug combinations**				
AD + AP	AD + AP + O	Other drug combinations†
6 (46.15%)	2 (15.38%)	5 (38.46%)

Changes in MADRS scores and osteocalcin levels are reported as percent changes related to the values in Week 0. Quantitative variable with not-normal distribution according to Shapiro-Wilk normality test (p < 0.05) was observed in osteocalcin change (*). **Comorbid diagnoses** are past diagnoses or a diagnosis not considered an exclusion criterion. Diagnostic codes of comorbidities are according to the International Classification of Diseases, 10th edition. F131 - Sedative, hypnotic, or anxiolytic-related abuse, F230 - Acute polymorphic psychotic disorder without symptoms of schizophrenia, F412 - Mixed anxiety and depressive disorder, F432 - Adjustment disorder, F531 - Severe mental and behavioral disorders associated with the puerperium, F601 - Schizoid personality disorder. Other drug combinations (†) include one patient with each of the following combinations: 2AD (two different antidepressants), 2AD + AP, AD + 2AP, AD + AP + MS, AD + 2AP + MS. BMI, body mass index; MADRS, Montgomery-Åsberg Depression Rating Scale; SD, standard deviation; IQR, interquartile range; AD, antidepressants; AP, antipsychotics; MS, mood stabilizers; ECT, electroconvulsive therapy; Other, other treatment including pregabalin.

### 3.2. Osteocalcin and depressive symptom severity

**Figure 1:**
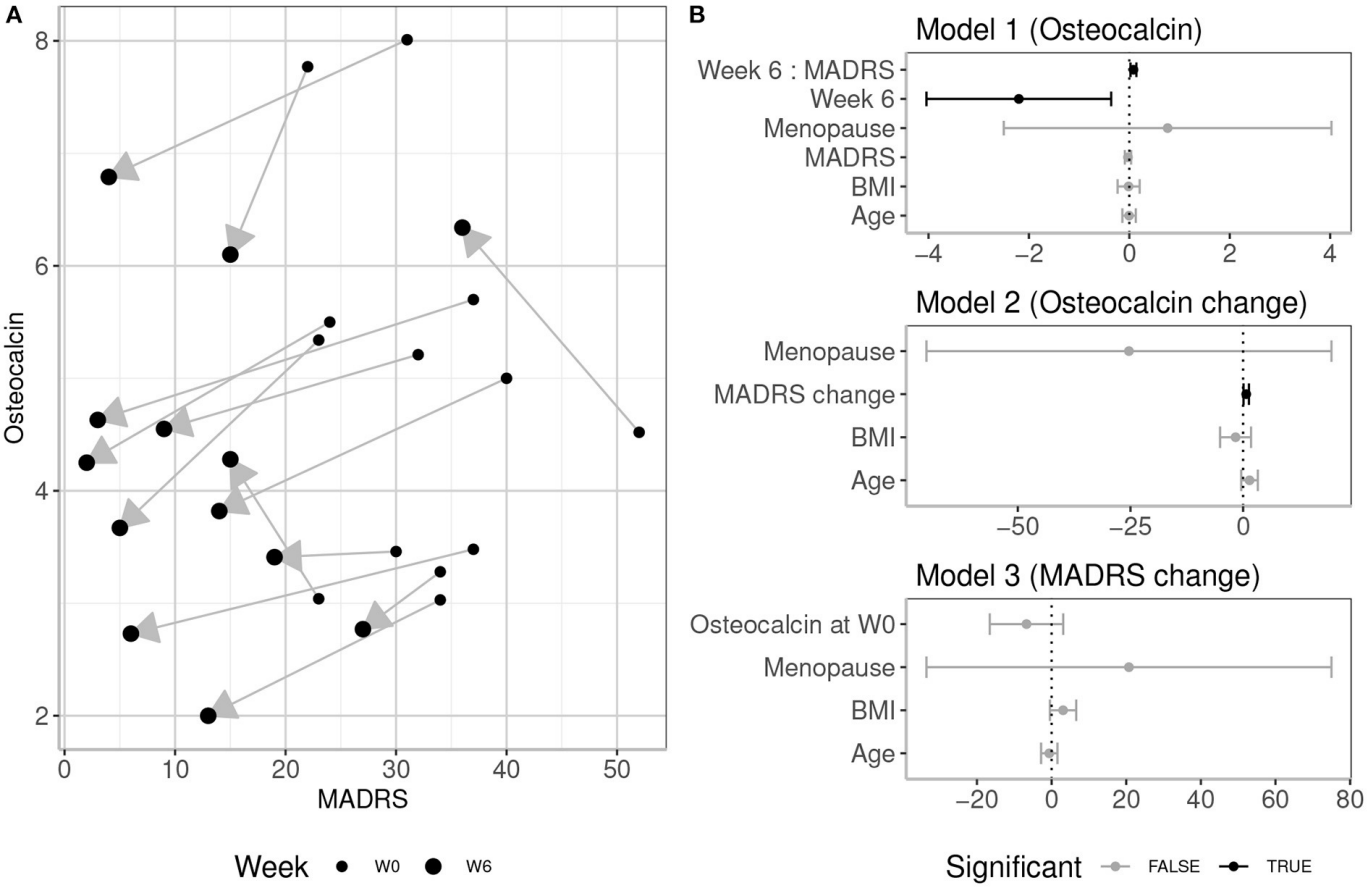
**(A)** MADRS scores and osteocalcin levels (ng/ml) at week 0 (W0) and week 6 (W6) in 13 subjects. Upward or downward direction of each arrow indicates individual increase or decrease in osteocalcin level, respectively. Of note is that in all but two subjects, the osteocalcin levels decreased. **(B)** 95% confidence intervals of effects in models used in analysis. Dependent variables of each model are indicated in parentheses. Interaction between variables is indicated by colon. Model 1 is a mixed-effects model, Models 2 and 3 are linear models. Statistically significant associations at p < 0.05 are depicted in black, non-significant in gray.

Changes in osteocalcin levels in relation to changes in MADRS, confidence intervals (CI) and model parameters are summarized in Table 3 and in Figure 1. In all but two subjects, the osteocalcin level decreased during 6 weeks of treatment. During the same period, the MADRS value decreased in all subjects. The mixed-effect model 1 did not reveal an association between osteocalcin and MADRS. However, osteocalcin levels were significantly lower at W6 than at W0 and there was a significant relationship between the osteocalcin levels and interaction of time and MADRS.

**Table 3:** Characteristics of models used in analyses.

**Model (DV)**	**Variable**	**t**	**p**	**95% CI**	**Partial η^2^**
Model 1	**Week 6**	**–2.345**	0.019*	**–4.041, –0.361**	**0.244**
(osteocalcin)	MADRS	–0.854	0.393	–0.089, 0.035	0.041
	Age	–0.071	0.942	–0.139, 0.129	<0.001
	Menopause	0.458	0.647	–2.501, 4.026	0.012
	BMI	–0.130	0.897	–0.234, 0.205	0.001
	**Week 6 : MADRS**	**2.734**	0.006*	**0.023, 0.141**	**0.305**
Model 2	**MADRS change**	**2.673**	0.028*	**0.095, 1.293**	**0.472**
(osteocalcin change)	Age	1.782	0.113	–0.419, 3.266	0.284
	Menopause	–1.3	0.23	–70.282, 19.612	0.174
	BMI	–1.118	0.296	–5.11, 1.773	0.135
Model 3	Osteocalcin W0	–1.577	0.153	-16.566, 3.111	0.237
(MADRS change)	Age	–0.661	0.527	–2.825, 1.566	0.052
	Menopause	0.881	0.404	–33.542, 74.995	0.088
	BMI	2.014	0.079	–0.445, 6.598	0.337

Model 1 is a mixed-effects model, Models 2 and 3 are linear models. Interaction between variables is indicated by the colon (:). Statistically significant results at p < 0.05 are bold and highlighted as *. Partial η^2^ is used as a measure of the effect size and can be interpreted according to Cohen (27): < 0.02 - very small, < 0.13 - small, < 0.26 - medium, > 0.26 - large. DV, dependent variable; CI, confidence interval; MADRS, Montgomery-Åsberg Depression Rating Scale; BMI, Body mass index.

Furthermore, although there was not a significant collective effect of MADRS change, age, menopausal status and BMI on osteocalcin change [*F*_(4, 8)_ = 2.366, *p* = 0.14, *R*^2^ = 0.313], linear model 2 showed a significant relationship between MADRS change and osteocalcin change.

The linear model 3 analyzing the relationship of osteocalcin in W0 and MADRS reduction did not uncover any significant collective or individual associations [*F*_(4, 8)_ = 1.637, *p* = 0.256, *R*^2^ = 0.175].

## 4. Discussion

The main finding of this study was that osteocalcin levels decreased during the first 6 weeks of treatment of depressive episode in the majority of patients and this decrease was associated with a reduction in depressive symptom severity.

Our results are in contrast with results from Aydin et al., who found am increase in osteocalcin levels during 3 months of escitalopram treatment of the first-episode major depression patients (22). This discrepancy may be due to the limited sample size in our study or different observation durations (3 months in study by Aydin et al., 6 weeks in our study). A possible effect of time on osteocalcin levels during the treatment of depression will be discussed further.

Our study included only hospitalized patients; there is no information about hospitalization in the study by Aydin et al., which was probably conducted in an outpatient or mixed setting (22). Considering that osteocalcin has a role in adaptation to exercise and increases with physical activity (29) our observation might have been related to lower physical activity during hospitalization. Lower sun exposure might have led to decreased vitamin D levels, which might have influenced our results further. On the other hand, it is questionable how daily activities and sun exposure differ in moderately to severely depressed out- and inpatients. Activity among outpatients with the major depressive disorder was shown to be objectively lower than activity among healthy individuals (30) and the relationship between sun exposure, vitamin D, and osteocalcin levels are not completely clear, as sun exposure may actually lower osteocalcin levels (31). This suggests that activity and sun exposure probably did not influence our results in a significant way.

Another possible explanation for our results is the direct effect of medication. Ortuño et al. reported a decrease in osteocalcin levels in male but not female mice after 6 weeks of fluoxetine administration, which was accountable for inhibiting serotonin signaling (32). However, in our study, patients used not only antidepressants but also antipsychotics. In that regard, the decrease in osteocalcin is in contrast with its increase in schizophrenia patients treated with antipsychotics (33) and it is, therefore, possible that decreases observed in our study were caused by other mechanisms.

That being said, neither the effect of inactivity or lower sun exposure due to hospitalization nor the effect of medication explain the observed association between osteocalcin decrease and symptom reduction. Recently it was reported that osteocalcin increases during acute stress responses (12) and it was demonstrated that its levels positively correlate with self-perceived stress in patients with depression (21) We, therefore, suggest that acute depressive episode is associated with significant stress, which is alleviated with effective treatment and is reflected by the decrease of osteocalcin levels.

Our results can also be explained with more direct effects of antidepressive treatment. Osteocalcin was proposed to increase monoamine synthesis in the brain (1); thus, we can hypothesize that osteocalcin decrease is a secondary effect of changes in monoaminergic transmission caused by anti-depressive medication, specifically monoamine-targeted antidepressants. In other words, there may be a feedback loop between osteocalcin release and monoaminergic activity in the brain, and this feedback loop may be mediated either by monoamines themselves, by glutamate (12) or other mediators. Interestingly, based on observation in mice, it has been suggested that there is a relationship between osteocalcin and brain neurotrophic factor (BDNF) expression (34). An increase in BDNF is often mentioned to accompany antidepressive treatment and alleviation of depressive symptoms; according to the meta-analysis by Zhou et al., there is indeed evidence that antidepressants may influence peripheral BDNF levels within a short amount of time and that different antidepressants elicit differential effects (35). This dependence of BDNF on time may be another explanation why our results contradict the study by Aydin et al. (22). Moreover, the relationship between BDNF and osteocalcin, both humoral factors regulating neuroplasticity and brain morphology in major depressive disorder, could be an intriguing focus of further research.

### 4.1. Limitations

This study has several limitations, notably the small sample size, and the inclusion of women of various ages with different menopausal status. However, menopausal status and age were accounted for in the models. The main result decreased osteocalcin levels, was observed consistently in all but two subjects; the probability that it is a spurious result is therefore low.

Patients used various drugs in various doses with various mechanisms of action which could have impacted the results. However, as already stated, most patients were administered antidepressants augmented with antipsychotics. Our results could therefore enable at least some degree of generalization for a subset of patients with relatively severe or resistant symptoms.

Another limitation is the lack of healthy controls and the resulting inability to examine differences between patients with depressive episodes and healthy individuals. The aim of the current study was, however, the relationship between osteocalcin levels, depression severity, and changes during treatment. For this purpose, the inclusion of healthy controls is not necessary.

Lastly, no sample size estimation was conducted prior to the data collection and analysis. It stems from the preliminary nature of this study and limited information about osteocalcin level changes during the treatment of depressive episode in current literature. Results of this study could be used to estimate sample size for future larger prospective studies.

### 4.2. Future directions

This study was conducted on a small sample of patients, and its results are only preliminary. However, considering that there is only a single similar work (22), this study demonstrated that osteocalcin might be related to depressive treatment response. It thus may lay the groundwork for further studies and the development of more complex methodologies. First, the inclusion of matched controls in a longitudinal study may help elucidate differences in osteocalcin levels between healthy individuals and patients with depression and the trajectory of its changes in time. Similarly, both males and females should be enrolled in future studies due to the known influence of sex on osteocalcin levels (4).

It has been observed that depression subtypes, e.g., melancholic or atypical, have relevance to another stress mediator, cortisol (36). Osteocalcin levels may be influenced correspondingly, and therefore better stratification of patients according to depressive symptom clusters would be helpful.

Because of the putative role of osteocalcin in GABA and monoaminergic neurotransmission (1), the specificity of our results to depression is also questionable: Are osteocalcin levels influenced by specific psychiatric symptoms, symptom severity, medication or do we observe only nonspecific reactions to stress? For that reason, osteocalcin in other psychiatric disorders and its relation to objective and subjective stress may be another topic to explore. Moreover, due to the distinct effects of antidepressant and antipsychotic medications on monoamines, better medication control is also needed.

Finally, it is possible that, similarly to other peripheral markers, plasma osteocalcin provides only a limited indication of its activity in the central nervous system. Some evidence of central osteocalcin effects may be obtained, for example, by assessing relationships between osteocalcin levels and brain morphology.

## 5. Conclusions

In this preliminary prospective study, osteocalcin decreased during the first 6 weeks of treatment of moderate and severe depressive episode in both pre- and postmenopausal women, and this decrease was associated with a reduction in depressive symptom severity. Because of major limitations, most notably small sample size, caution is needed to draw any definitive conclusions and generalize results. However, this study suggests that the utility of osteocalcin as a biomarker of depressive episode treatment response warrants further investigation in larger studies with better methodologies.

## Data availability statement

The raw data supporting the conclusions of this article will be made available by the authors, without undue reservation.

## Ethics statement

The studies involving human participants were reviewed and approved by Ethics Committee of University Hospital Brno, University Hospital Brno, Czech Republic. The patients/participants provided their written informed consent to participate in this study.

## Author contributions

EB contributed to the data analysis and authored a first draft of the text, figure, and tables. JH contributed to the design of the study, performed the literature gathering, and authored portions of the text, especially the introduction section. PK contributed to the patient recruitment, clinical assessment, data gathering, and participated in writing the text, especially the discussion section. AD contributed to the clinical assessment, patient data gathering, and participated in writing the text. JT, MT, JF, and JK performed the osteocalcin analysis, participated in data analysis, contributed reagents, materials, and analysis tools. JB-V supervised the study, provided expertise on osteocalcin and stress response, and supervised the writing of the text. All authors contributed to the article and approved the submitted version.

## Funding

This work was supported by a grant from the Ministry of Health of the Czech Republic (FNBr, 65269705). The project was supported also by the CETOCOEN PLUS (CZ.02.1.01/0.0/0.0/15_003/0000469) project of the Ministry of Education, Youth and Sports of the Czech Republic and by CETOCOEN EXCELLENCE Teaming 2 project supported by Horizon2020 [857560] and the Ministry of Education, Youth and Sports of the Czech Republic (02.1.01/0.0/0.0/18_046/0015975), as well as by the RECETOX Research Infrastructure (LM2018121).

## Conflict of interest

Author EB declares a commercial relationship with Angelini S.p.a. and H. Lundbeck A/S in the form of honoraria. The remaining authors declare that the research was conducted in the absence of any commercial or financial relationships that could be construed as a potential conflict of interest.

## Publisher's note

All claims expressed in this article are solely those of the authors and do not necessarily represent those of their affiliated organizations, or those of the publisher, the editors and the reviewers. Any product that may be evaluated in this article, or claim that may be made by its manufacturer, is not guaranteed or endorsed by the publisher.
